# Potential Adverse Consequences of Combination Therapy with Gabapentin and Pregabalin

**DOI:** 10.1155/2021/5559981

**Published:** 2021-06-02

**Authors:** Muhammad Nabeel Ghayur

**Affiliations:** ^1^Kentucky College of Osteopathic Medicine, University of Pikeville, Pikeville, KY 41501, USA; ^2^Registered Pharmacist, Community Pharmacy, Ontario, Canada

## Abstract

Gabapentinoids comprise the medications gabapentin and pregabalin. These were designed to not only look chemically like the central inhibitory neurotransmitter gamma-aminobutyric acid (GABA) but also act like it. The prototype gabapentin was primarily introduced to be used as antiepileptic medication. Today, both chemicals are not only utilized as adjunct antiepileptics in focal (aware and impaired awareness) seizures but are also used in several neuropathic pain conditions and other clinical indications. Their use has skyrocketed in the past few years and this has brought forward more instances of adverse effects and errors in prescribing practices. We describe here a case of a female patient with a history of diabetes, diabetic neuropathy, and hypertension being prescribed both gabapentin and pregabalin concomitantly which led to adverse effects like drowsiness, dizziness, fatigue, and ataxia. Once the patient medication profile was revisited, the pharmacy staff was able to identify the therapeutic duplications (gabapentin and pregabalin). The physician was contacted and pregabalin was discontinued. This led to the disappearance of the adverse effects. The dose of the existing gabapentin was increased to control the symptoms of diabetic neuropathy. This report sheds light on the importance of responsible prescribing, efficient checking of medication profiles on the level of dispensing pharmacies, and timely follow-up to patients to keep the patients safe and their medical conditions under check.

## 1. Introduction

**Figure 1:**
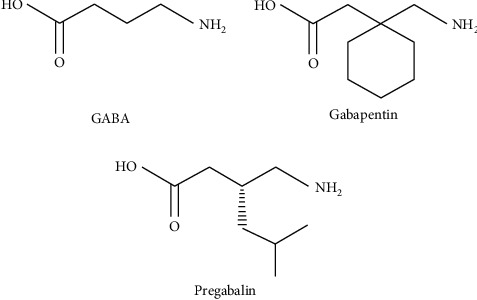
Chemical structures for gamma-aminobutyric acid (GABA) and the gabapentinoids, first-generation compound gabapentin and second-generation pregabalin.

Gabapentinoids are a group of chemicals comprising gabapentin and pregabalin. These are the chemical structure derivatives of the inhibitory neurotransmitter, gamma-aminobutyric acid (GABA). Both agents include in their structure (Figure 1), a GABA molecule plus a lipophilic cyclohexane ring (gabapentin) or isobutane (pregabalin). These agents were introduced to not only look like GABA but also act like GABA, highly lipid-soluble to cross the blood-brain barrier [1].

In 1993, the US Food and Drug Administration (FDA) approved the prototype gabapentin, a first-generation gabapentinoid, for the management of seizures. Today, it is used as a second-line/adjunct agent in the therapy of focal (aware and impaired awareness) seizures. Later, gabapentin was also approved for use in pain control of postherpetic neuralgia (Table 1). The second-generation gabapentinoid, pregabalin, was introduced in 2004 and today enjoys several labelled clinical indications including focal seizures and neuropathic pain [2], as shown in Table 1. Around 4% of the US population used these prescription gabapentinoids each year [3], indicating the widespread consumption of these medications.

Gabapentinoids primarily act via binding to a protein, called the *α*2*δ* subunit (or the “gabapentin receptor”), on central voltage-dependent Ca^2+^ channels [1]. The exact mechanism of the anticonvulsant, analgesic, anxiolytic, and sleep-enhancing properties of gabapentinoids is still unclear. However, there are indications that the binding leads to a reduction in the release of excitatory neurotransmitters such as glutamate, norepinephrine, calcitonin gene-related peptide (CGRP), and substance P [4].

The widespread use of gabapentinoids has not been without issues and negative consequences. Among the abuse potential of the agents, there is also the occurrence of adverse effects, as it is with any other medication. The main adverse effects (in >10% of consumers) for both gabapentin and pregabalin are ataxia, drowsiness, dizziness, and fatigue [5, 6]. Around one-third of the patients taking these would experience dizziness and somnolence [7, 8].

In this communication, we report a case with a patient experiencing adverse effects while being on both gabapentinoids at the same time for diabetic neuropathy. Pharmacist intervention led to the discontinuation of one of the agents and the subsequent prevention of the major consequences of this combination.

## 2. Case Report

A 57-year-old female patient presented to the community pharmacy complaining of fatigue, drowsiness, and dizziness for the past 2 weeks. The patient had a medical history of type 2 diabetes mellitus and hypertension. She was on metformin 500 mg three times a day, gliclazide MR 30 mg twice daily, ramipril 5 mg once daily, and gabapentin 100 mg three times a day and was recently started on pregabalin 25 mg twice a day. She was also on a multivitamin tablet once daily, vitamin D 1000IU 2 tablets once daily, and a vitamin B12 1000mcg tablet once daily. She had no allergies to medication, did not smoke tobacco, only drank alcohol socially once or twice a month, and had never used recreational drugs. The patient's blood pressure (BP) and blood glucose (BG) levels were in control. BP reading in the pharmacy was 131/78 mm/Hg while the last HB1AC a month ago was 6.8%.

The patient was diagnosed with diabetes 5 years ago. She was started on metformin first and then consequently gliclazide was initiated to control the out-of-range BG levels. Due to the known long-term microvascular complications of diabetes, the patient started experiencing loss of sensation in the periphery and numbness and tingling in the hands and feet. To rectify these symptoms, she was started 3 months ago on gabapentin 100 mg once daily at bedtime for a week, with an option to increase the dose by 100 mg per week up to 100 mg three times daily. Although gabapentin helped in the beginning, later patient saw a resurgence of her diabetic neuropathy symptoms. In response to this, the patient's primary care physician started her on a second gabapentinoid pregabalin at 25 mg once daily at bedtime with the option to increase the dose by 25 mg weekly to a maximum of 50 mg per day. The patient was taking both these gabapentinoids concomitantly, gabapentin 300 mg per day and pregabalin 50 mg per day. This caused the patient to experience drowsiness, fatigue, dizziness, and even ataxia.

The patient's profile was revisited in the pharmacy as per the issues that were being faced by the patient. The pharmacist confirmed all prescription and nonprescription medications. Neither there were any new medical conditions, nor the BP or BG levels were found out of the range that could have resulted in fatigue and other symptoms (an increased effect from antihypertensive and antidiabetic medications could have resulted in these symptoms). The only change was the addition of pregabalin to the patient's drug regimen. A good therapeutic tool would have been the gabapentin and pregabalin serum levels. These were not available, but if they were, these could have directly indicated the cause of the symptoms to the increased levels of the gabapentinoids. For gabapentin, a serum concentration range of 2–20 mg/l is linked to a therapeutic effect while for pregabalin, an approximate range of 2.8–8.3 mg/l is suggested [9]. As the patient started to get diabetic neuropathy symptoms again, the physician, rather than increasing the dose of existing gabapentin, started the patient on pregabalin, thus resulting in therapeutic duplication. The error was also not picked up by the pregabalin dispensing pharmacist, and the patient was left to deal with the adverse consequences. Later, after noticing this error, the pharmacist contacted the physician, got the pregabalin discontinued, and requested for the dose of gabapentin to be increased from 100 mg three times a day to 100 mg in the morning, 100 mg in the afternoon, and 200 mg at bedtime with the option to increase by 100 mg weekly to a maximum of 200 mg three times a day. This intervention helped the patient as she got rid of fatigue, drowsiness, and dizziness, while her neuropathy symptoms also improved with the increased dose of gabapentin.

## 3. Discussion

An estimated 2.4% (around 8 million people) of the US population suffers from peripheral neuropathy, while 25% of the diabetic cohort suffers from diabetic neuropathy [10]. Gabapentinoids are regarded as first-line agents to prescribe for this kind of pain. In the US alone, for gabapentin, the number of prescriptions increased by 64% from 2012 to 2016. Therefore, in 2017, around 70 million scripts of gabapentin were processed, making it one of the top 10 medications in the country [11].

The mechanism of action of gabapentinoids revolves around the fact that they can exhibit CNS depression via inhibition of central voltage-gated Ca^2+^ channels which leads to a reduction in the levels of excitatory neurotransmitters like glutamate, norepinephrine, and other nociceptive and neuroinflammatory neurotransmitters like CGRP and substance P [4, 12]. Therefore, most probably, these medications are used as antiepileptics and analgesics, particularly in pain of neuronal origin [1, 12]. In addition to this, gabapentin and pregabalin are also used in several other off-label indications such as cough, anxiety, pruritis, restless leg syndrome, and menopause (for gabapentin and pregabalin) while gabapentin is also used in alcohol withdrawal, fibromyalgia, hiccups, and depression [5, 6]. Their use has increased owing to the scarcity of options for pain control and tougher restrictions on prescribing opioids for noncancer pain [2]. Although with such a variety of analgesic uses, their clinical benefits are yet to be proven in clinical trials [13].

This does not mean they come without any adverse reactions. And these adverse reactions are compounded in a situation when a patient is prescribed both these agents concomitantly. Both these medications are central nervous system (CNS) depressants and one can imagine the typical and strong reaction a person might get if put on both at the same time. When a drug-drug interaction analysis for gabapentin and pregabalin is done, it reveals that taking both these CNS depressants can lead to adverse effects like (but not limited to) ataxia, confusion, drowsiness, respiratory depression, and weakness [14]. This is exactly what this patient experienced. However, stopping pregabalin helped mitigate the situation and the patient's adverse effects went away. Postdiscontinuation of pregabalin, the dose of gabapentin was also increased to give the patient relief from the lingering symptoms of neuropathy.

Gabapentin is different from pregabalin in terms of pharmacokinetics (Table 1) in a way that with gabapentin, there is a saturation of its absorption while pregabalin has linear kinetics. Although this was not a problem with this patient because she was on a comparatively smaller dose of gabapentin to start on and rather than adding pregabalin, the dose of gabapentin could have easily been increased for clinical benefit, which was done later. Gabapentin can be given till 1800 mg daily as after that there is a minimal benefit while more chance of getting adverse effects [15]. Even if she were on both medications together, there is no benefit of taking them together because pregabalin has a six times higher affinity for binding to the *α*2*δ* subunit/gabapentin receptor and would have prevented gabapentin from binding [16, 17]. Not to mention that both medications also are eliminated via the kidneys (Table 1) so that would be a burden on the kidneys too, particularly if the patient had a type of renal dysfunction. There have been a few studies in the literature looking at combination therapy with gabapentin and pregabalin [18, 19]. Although as discussed earlier, this would require careful monitoring and oversight from healthcare providers so that the combined CNS depression from both medications does not lead to serious consequences of gait leading to falls and fractures particularly in the elderly [20].

Lastly, one more important point to consider in controlling the way these gabapentinoids are prescribed is their abuse and misuse potential [2, 11, 13]. Along with the abovementioned central adverse effects, these medications can be addictive, particularly in people with other addictions or when combined with other drugs of abuse particularly opioids [21], although the risk is low. Rare cases of overdose deaths have also been reported from gabapentin and pregabalin [22]. Gabapentinoids can produce euphoria, dissociation, and other psychedelic effects [11]. They also produce withdrawal symptoms so should not be stopped abruptly but tapered off slowly over a period of weeks [2]. All of these aspects of caution should particularly be incorporated in prescribing practices and patient education by the prescribers. At the time of initial prescribing, each and every patient should be assessed for appropriateness of therapy. If the patient is prone to adverse effects, then other therapeutic tools should be utilized. Patients should be periodically monitored for mental alertness, sedation, respiratory depression, and renal function [6]. Patients should also be advised not to stop the medication themselves as a careful tapering schedule is needed under the supervision of the prescriber.

This report not only focuses on the issue of therapeutic duplication, CNS depressant adverse effects, and consequences of potential combined gabapentin and pregabalin therapy but also highlights the importance of responsible prescribing particularly with centrally acting/psychoactive medications. Although this is a requirement for any kind of medication, chemicals that can cause harm and can be addictive pose more danger. This communication also reiterates the importance of double-checking drug-drug interactions, therapeutic duplications, and patient medication profiles at the level of community pharmacies so that errors like these are caught promptly and corrected with the consent and approval of prescribers. This way, medication errors are prevented, and patients are provided effective health care.

## 4. Conclusion

The intention of this case report was to increase awareness about the adverse effects of these widespread medications. With increased use, there are increased instances of adverse effects, and therefore, it is the prime responsibility of healthcare professionals to make the best clinical judgement in selecting the right medications. Gabapentin and pregabalin are being massively prescribed for neuropathic pain of all kinds of etiologies. However, if these medications are combined or if given in a high dose, they can lead to adverse effects such as dizziness, drowsiness, fatigue, weakness, and somnolence. These medications also carry the risk of addiction, which makes it even more important to control their prescribing.

## Figures and Tables

**Table 1:** Comparison of pharmacokinetic properties and US FDA-labelled indications of gabapentin and pregabalin.

Characteristics	Gabapentin	Pregabalin
Administration	Oral, parenteral (for misuse)	Oral (parenteral for misuse)
Absorption	Oral: saturable	Oral: not saturable (linear)
	Parenteral: linear	Parenteral: linear
Bioavailability	27–60% (inversely proportional to dose)	≥90%

Dose frequency	TID	BID
Metabolism	Negligible	Negligible
Excretion	Urine (unchanged)	Urine (unchanged)
Indication (label)	Focal seizure, postherpetic neuralgia	Focal seizure, postherpetic neuralgia, fibromyalgia, neuropathic pain (diabetes, spinal cord injury)

US FDA: United States Food and Drug Administration; TID: three times a day; BID: twice a day.

## Data Availability

No data were used to support this study.
